# Short-range Lidar SLAM utilizing localization data of monocular localization

**DOI:** 10.1186/s40648-021-00211-7

**Published:** 2021-10-26

**Authors:** Sousuke Nakamura, Shunsuke Muto, Daichi Takahashi

**Affiliations:** 1 Faculty of Science and Engineering, Hosei University, 3-7-2, Kajino-cho, Koganei, Tokyo 184-8584 Japan; 2 Graduate School of Science and Engineering, Hosei University, 3-7-2, Kajino-cho, Koganei, Tokyo 184-8584 Japan

**Keywords:** SLAM, Sensor fusion, Calibration

## Abstract

Simultaneous localization and mapping (SLAM) is a widely used technology in autonomous mobile robots, where sensors such as Lidar or cameras are typically used. Sensor fusion using multiple sensors has been employed to compensate for the shortcomings of each sensor in SLAM. However, the sensor cost cannot be ignored when considering its practical usage. Therefore, this study aims at realizing a high-precision SLAM using a sensor switching system, combining multiple low-cost sensors. The sensor switching system consists of a low-cost Lidar SLAM and a monocular localization. Since a low-cost Lidar has a short laser range, degeneracy often occurs due to the fact that they cannot capture features while building maps. The proposed system uses localization data from monocular localization to ensure precision in regions where degeneracy occurs. The proposed system was evaluated through the simulation assuming the museum environment where the degeneracy occurred. The accuracy of the robot trajectory and the built map proved the effectiveness of the proposed system.

## Introduction

Autonomous mobile robots are used in various environments and applications, such as transport robots in factories and service robots in facilities. These robots can solve the labor shortage issue caused by the declining birth rate and aging population, save labor, and improve efficiency by automating tasks. In recent years, due to the COVID-19 pandemic, the demand for autonomous mobile robots that can replace human labor is expected to increase. In addition, a more accurate movement of the robots and a reduction in the installation costs are expected.

A high-accuracy simultaneous localization and mapping (SLAM) system [1] is required for stable and accurate autonomous movement in practical applications. Furthermore, low-cost sensors are desirable for SLAM. Typical sensors used in SLAM are Lidar and cameras.

Lidar can easily realize high-accuracy SLAM as they capture the distance to the surrounding environment by laser irradiation to acquire point cloud data of the surrounding environment. However, Lidar with a wide range (hereinafter referred to as long-range Lidar) is expensive.

In contrast, low-cost cameras can acquire information over long distances because they identify the surrounding environment based on information available through images. However, a monocular camera cannot directly measure the distance to the surrounding environment; thus, it is necessary to calculate the distance, which contributes to the inaccuracy of SLAM. RGB-D cameras can acquire depth information; however, the accuracy is lower than that of Lidar and is more expensive than monocular cameras.

**Fig. 1:**
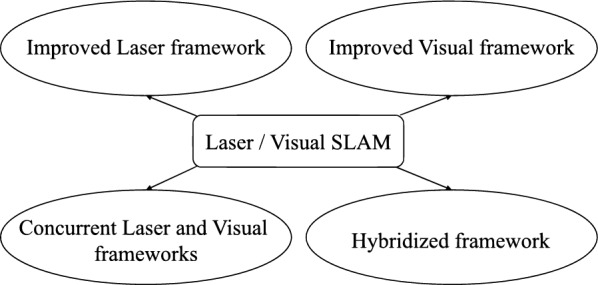
Various approaches of implementing Laser-Visual SLAM [15]

Because each sensor has advantages and disadvantages, sensor fusion has been used in SLAM. In particular, many studies performed sensor fusion between Lidar and cameras [2–14], classified as shown in Fig. 1 [15]. Although there are some studies that used cameras other than monocular cameras [2–6], this paper presents related studies focused on sensor fusion using a Lidar and a monocular camera.

Some studies improved the SLAM performance with a monocular camera (hereinafter referred to as monocular SLAM) using Lidar. For example, in [7–9] the problem of uncertain map scale in monocular SLAM was solved using lasers. In contrast, several studies improved the performance of Lidar SLAM using a monocular camera. For example, focusing on the fact that the monocular camera has a high environment recognition performance, there are some studies that utilized the visual information of the camera for loop detection and scan matching in Lidar SLAM [10–13].

In addition, as an approach similar to the authors work described below, a study utilized the pose information from the monocular SLAM [14]. In reference [14], a calibration method that focuses on the robot trajectory is applied to the monocular SLAM, and therefore, could use the pose information of the monocular camera when Lidar SLAM is degraded. However, this method was based on the assumption that the map built by SLAM is accurate to a certain extent; this method can not be used in supplementing the poses in real time during SLAM. Therefore, when degeneracy occur during the map build, the accuracy of the map built by Lidar SLAM is degraded, which results in poor accuracy of the calibration; the supplement by the pose information of the monocular camera will not function accurately. Nevertheless, an approach to actively use Lidar with a narrow range (hereinafter referred to as “short-range Lidar”) is quite practical in terms of reducing the cost.

This study proposes a low cost yet highly accurate Lidar SLAM under following two conditions: (1) Limited to extremely low-cost sensor configuration of short-range 2D Lidar and monocular camera, (2) Assume an indoor space where monocular SLAM is basically well performed but short-range Lidar SLAM degenerates in some areas.

Specifically, this is a method that maintains the accuracy of a short-range 2D Lidar SLAM operated as a base, by using the pose information from the monocular SLAM as the supplement in case the degeneracy is automatically detected. In this respect, the proposed method is a sensor switching method rather than a fusion in terms of localization.

Degeneracy refers to a situation in which scan-based pose estimation, such as scan matching [16], is not accurately performed. Degeneracy is more likely to occur in short-range Lidar with a narrow scan range because it is difficult to capture the features needed for posing. When degeneracy occurs, the robot misunderstand that a scan was obtained at the same pose even if the robot is moving; thus, a map that is shrunk in the direction of translation compared to the real environment is obtained. Although it is possible to suppress degeneracy to certain extent by using odometry, the accuracy of odometry cannot be ensured because of the cumulative error.

The structure of this paper is as follows. In “Localization supplement system by monocular localization”, the authors propose a system that utilizes the pose information of the monocular localization in case the degeneracy occurs to maintain the accuracy of mapping build of the short-range Lidar SLAM. In “Simulation”, the performance of the proposed system is evaluated through simulations. Finally, in “Conclusion”, a summary of the study and future works are described.

## Localization supplement system by monocular localization

### System overview

**Fig. 2:**
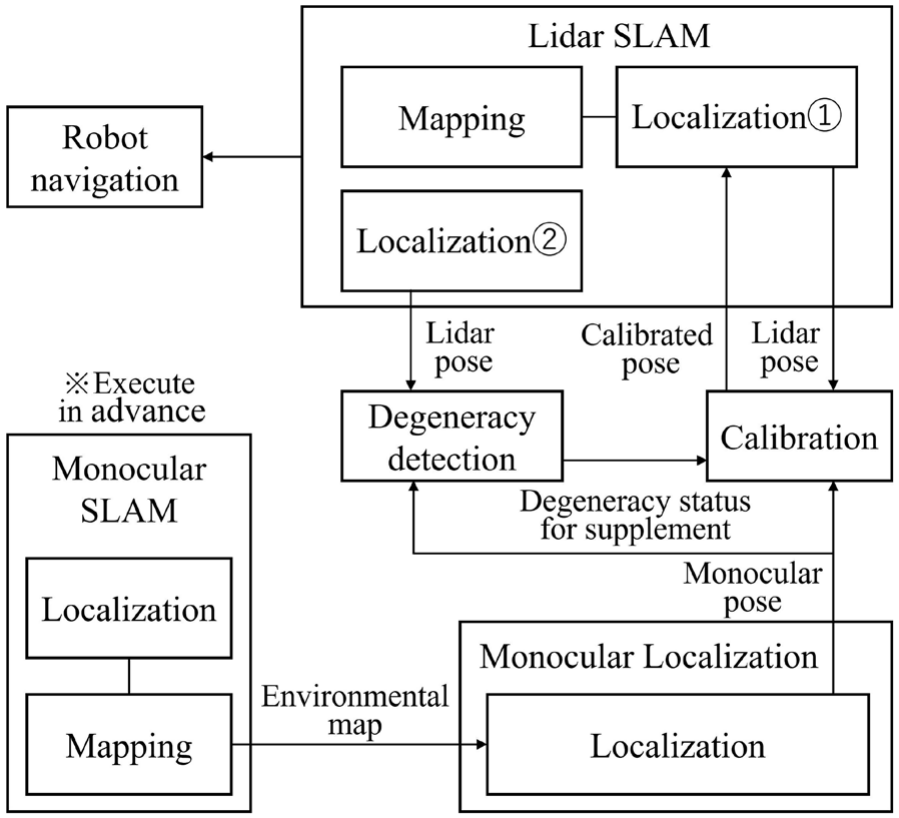
Proposed system

Figure 2 shows the configuration of the proposed system. The goal of the system is to generate the accurate map based on the short-range Lidar SLAM which could be adequately used for autonomous navigation of the robot afterwards, even in case the degeneracy occurs. Thus, in environments where degeneracy occurs in short-range Lidar SLAM, the system considers using the pose information from monocular localization, after applying the coordinate system calibration method described below, instead of the pose information from Lidar SLAM. Figure 2 shows that the proposed system operates based on the short-range Lidar SLAM, and the calibrated pose from monocular localization is prepared as a substiture for Lidar pose in case the degeneracy occurs. It should be noted that this calibrated pose is automatically caliculated in realtime during the SLAM. In this sense, the proposed system could be categorized as the improved Lidar framework which improves the accuracy of Lidar SLAM owing to the use of camera in localization, as shown in Fig. 1. The details of the algorithm is as follows.

### Algorithm

**Fig. 3:**
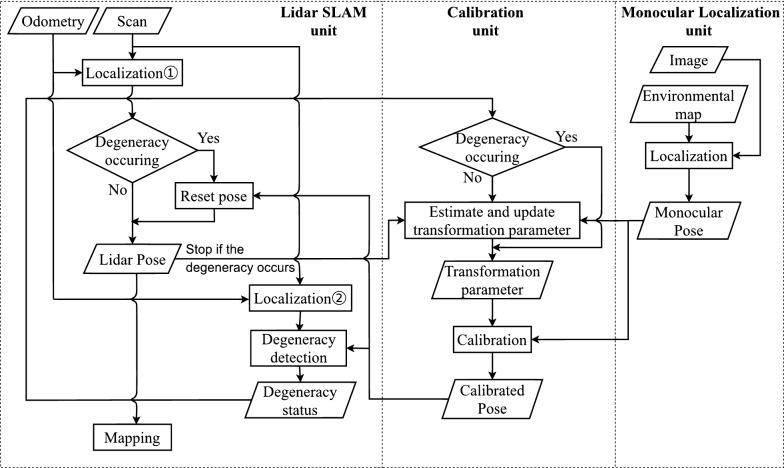
Detailed system flowchart

The algorithm of the proposed system is shown in Fig. 3. The proposed system can be divided into three units corresponding to Lidar SLAM, monocular localization, and calibration. Each unit is processed parallelly, and the pose information from the Lidar SLAM (Lidar pose), monocular localization (monocular pose), calibrated monocular localization (calibrated pose), and degeneracy detection information is transmitted and received between each unit. In the Lidar SLAM unit, scan and odometry are acquired, and scan matching is used to estimate the pose. In case degeneracy occurs, the calibrated pose is set to the Lidar pose (see Localization 1 of Figs. 2, 3) and used for map construction. On other hand, when degeneracy does not occur, the map is built based on the pose information obtained by scan matching.

The degeneracy detection is performed by focusing on the fact that the displacement of the pose is under estimated in the direction of translation or rotation when degeneracy occurs. In detail, the amount of displacement of the Lidar pose and the calibrated pose from previous time step to the present time step is calculated respectively, and in case their difference is larger than a threshold value, the degeneracy is assumed to be detected. However, since the calibrated pose is set to the Lidar pose in case degeneracy occurs as mentioned above (see Localization 1 of Figs. 2, 3), the problem that raw Lidar pose can not be used for the degeneracy detection arise. To deal with this problem, the raw Lidar pose which is not overwritten in any case (see Localization 2 of Figs. 2, 3) is also prepared and it was used for degeneracy detection.

Thus, the degeneracy detection based on the Lidar pose (of Localization 2) and the calibrated pose at the timing when the robot acquires the *k* th pose is expressed as follows. The translation distance is calculated by Eqs. (1) and (2), and the rotation distance is calculated by Eqs. (3) and (4), using $(x_k^l,y_k^l,\theta _k^l)$ and $(x_k^c,y_k^c,\theta _k^c)$, respectively. Here, $k-1$ represents the information from previous time step.

1 $$\begin{aligned} r_k^l= \sqrt{(x_k^l)^2+(y_k^l)^2}-\sqrt{(x_{k-1}^l)^2+(y_{k-1}^l)^2} \end{aligned}$$

2 $$\begin{aligned} r_k^c= \sqrt{(x_k^c)^2+(y_k^c)^2}-\sqrt{(x_{k-1}^c)^2+(y_{k-1}^c)^2} \end{aligned}$$

3 $$\begin{aligned} \phi _k^l= \theta _k^l-\theta _{k-1}^l \end{aligned}$$

4 $$\begin{aligned} \phi _k^c= \theta _k^c-\theta _{k-1}^c \end{aligned}$$

Then, the past n times average of the difference in travel distances between the Lidar pose and the calibrated pose is calculated, as shown in Eq. (5) and Eq. (6).

5 $$\begin{aligned} \Delta r_k= \frac{1}{n}\sum _{i=0}^{n-1}|r_{k-i}^l-r_{k-i}^c| \end{aligned}$$

6 $$\begin{aligned} \Delta \phi _k= \frac{1}{n}\sum _{i=0}^{n-1}|\phi _{k-i}^l-\phi _{k-i}^c| \end{aligned}$$

In case the estimated travel distance derived by the Lidar pose becomes smaller due to degeneracy, there will be a certain difference from that derived by calibrated pose. Therefore, when $\Delta r_k$ in Eq. (5) or $\Delta \phi _k$ in Eq. (6) becomes larger than a certain value, it indicates that degeneracy has occurred. Note that we applied a quite simple degeneracy detection method in this study and, if necessary, methods such as [17] and [18] could be applied to improve the performance.

In this paper, the pose of n times is required for the degeneracy detection, and there is a difference between the time when robot entered the degenerate environment and the time when the detection is made. This leads to using inaccurate pose while this n times delay. To solve this problem, the calibrated pose is always stored up to n time steps ago, and when the detection result switches from “non-degenerate” to “degenerate”, the Lidar pose is reset up to n time steps ago with these stored calibrated poses.

The monocular localization unit estimates the robot’s pose from the acquired image and transmits the monocular pose to the calibration unit. In this case, the monocular SLAM also prepares and executes the map in advance.

The calibration unit obtains the degeneracy detection information from the Lidar SLAM unit. If degeneracy does not occur, the estimated values of the transformation parameters are updated using the Lidar pose obtained from the Lidar SLAM unit and monocular pose obtained from the monocular localization unit according to the coordinate calibration method described below. In contrast, if degeneracy occurs, the estimated values of the transformation parameters are not updated. Finally, the estimated transformation parameters are applied to the monocular pose to derive the calibrated pose, which is transmitted to the Lidar SLAM unit.

### Coordinate calibration method

The coordinate systems of the map built by Lidar SLAM and monocular SLAM are different. A monocular camera cannot estimate the physical distance due to its structure and is processed as a dimensionless quantity. Moreover, the origin and direction of each axis of the coordinate systems depend on pose of the sensors with respect to the robot. Hence, calibration between the coordinate systems is required in order to convert the monocular pose into the Lidar pose.

In this study, an automatic calibration method that uses only the robot’s pose information is developed based on practicality. Although several coordinate calibration methods have been currently proposed [14, 15, 19–21], an automatic calibration method was developed referring to the method [21] and adding a scale estimation function to it.

**Fig. 4:**
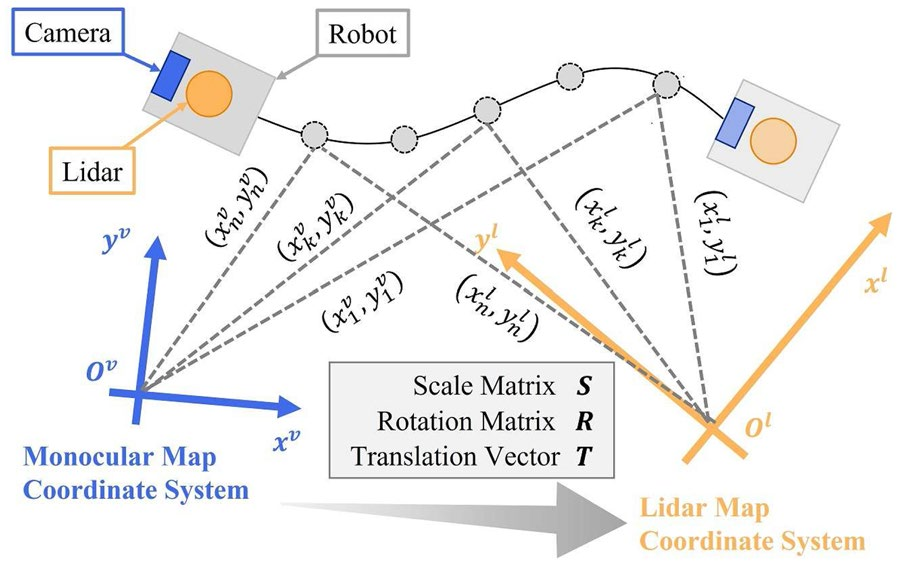
Conceptual image of automatic calibration utilizing robot trajectory in two coordinate systems

A visualization of the automatic calibration is shown in Fig. 4. The coordinate systems of Lidar map and monocular map are represented as $O^l$ and $O^m$, and the position coordinates by $(x^l,y^l )$ and $(x^m,y^m )$, respectively. The robot trajectory in each coordinate system was recorded to automatically estimate the transformation parameters between each coordinate system. The transformation parameters were estimated using the same pose coordinates of the robot obtained independently by Lidar SLAM and monocular localization.

Here, the calibration from the monocular map coordinate system to the Lidar map coordinate system is considered. Using the monocular map coordinate system, the Lidar map coordinate system can be expressed as Eq. (7) with *S* as the scaling matrix, *R* as the rotation matrix, and *T* as the translation vector.

7 $$\begin{aligned} O^l=SRO^m+T \end{aligned}$$

Therefore, in detail, the Lidar map coordinate system has a relation with the pose coordinates of the monocular map coordinate system as shouwn in Eq. (8).

8 $$\begin{aligned} {\displaystyle \begin{aligned} \begin{pmatrix} x^l \\ y^l \\ \end{pmatrix}=\begin{pmatrix} S_x^{lm} &{} 0 \\ 0 &{} S_y^{lm} \\ \end{pmatrix}\begin{pmatrix} \cos \theta ^{lm} &{} -\sin \theta ^{lm} \\ \sin \theta ^{lm} &{} \cos \theta ^{lm} \\ \end{pmatrix}\begin{pmatrix} x^m \\ y^m \\ \end{pmatrix}+\begin{pmatrix} T_x^{lm} \\ T_y^{lm} \\ \end{pmatrix} \end{aligned}} \end{aligned}$$

where $S_x^{lm}$ and $S_y^{lm}$ represent the scaling in the *x* and *y* directions, respectively; $\theta ^{lm}$ represents the rotation direction; $T_x^{lm}$ and $T_y^{lm}$ represent the translation in the *x* and *y* directions, respectively. Here, the transformation parameters are estimated by the least-squares method shown in Eq. (9) [19], where $(x_k^l,y_k^l )$ and $(x_k^m,y_k^m )$ represent the poses of the *k* robot in each coordinate system of Lidar map and monocular map, respectively.

9 $$\begin{aligned} {\displaystyle \begin{aligned} \varepsilon ^2&=\sum _{k=1}^{n}\{(x_k^l-S_x^{lm}x_k^m\cos \theta ^{lm}+S_x^{lm}y_k^m\sin \theta ^{lm}-T_x^{lm})^2 \\&\quad \quad +(y_k^l-S_y^{lm}x_k^m\sin \theta ^{lm}-S_y^{lm}y_k^m\cos \theta ^{lm}-T_y^{lm})^2\} \end{aligned}} \end{aligned}$$

If two or more pairs $(n\ge 2)$ of corresponding points are obtained from the previous robot trajectory, $\frac{\partial \varepsilon ^2}{\partial T_x^{lm}}=0$, $\frac{\partial \varepsilon ^2}{\partial T_y^{lm}}=0$, $\frac{\partial \varepsilon ^2}{\partial S_x^{lm}}=0$, $\frac{\partial \varepsilon ^2}{\partial S_y^{lm}}=0$, $\frac{\partial \varepsilon ^2}{\partial \theta ^{lm}}=0$ becomes valid and the transformation parameters can be estimated in principle by solving this system of equations. However, this nonlinear system of equations cannot be solved analytically and can be computed iteratively using the Newton method. Therefore, the approximate solutions are obtained using Eq. (12), where the *i*-th approximate solution is Eq. (10) and the correction values is Eq. (11).

10 $$\begin{aligned}&P_i= \left( \begin{array}{ccccc} T_{x,i}^{lm}&T_{y,i}^{lm}&S_{x,i}^{lm}&S_{y,i}^{lm}&\theta _i^{lm} \end{array} \right) ^{\mathrm {T}} \end{aligned}$$

11 $$\begin{aligned}&{\displaystyle \begin{aligned} \Delta P= \left( \begin{array}{ccccc} \Delta T_{x}^{lm}&\Delta T_{y}^{lm}&\Delta S_{x}^{lm}&\Delta S_{y}^{lm}&\Delta \theta ^{lm} \end{array} \right) ^{\mathrm {T}} \end{aligned}} \end{aligned}$$

12 $$\begin{aligned}&P_{i+1}=P_i+\Delta P \end{aligned}$$

Here, the appropriate initial value is set to $P_0=(T_{x,0}^{lm}$, $T_{y,0}^{lm}$, $S_{x,0}^{lm}$, $S_{y,0}^{lm}$, $\theta _0^{lm})$, and the correction value $\Delta P$ is calculated by Eq. (13), which is derived from Newton’s method. Y and X of Eq. (13) are calculated using Eq. (14) and (15), respectively.

13 $$\begin{aligned}&\Delta P = Y^{-1}(-X) \end{aligned}$$

14 $$\begin{aligned}&Y=\left( \begin{array}{ccccc} \frac{\partial A}{\partial T_x^{lm}} &{} \frac{\partial A}{\partial T_y^{lm}} &{} \frac{\partial A}{\partial S_x^{lm}} &{} \frac{\partial A}{\partial S_y^{lm}} &{} \frac{\partial A}{\partial \theta ^{lm}} \\ \frac{\partial B}{\partial T_x^{lm}} &{} \frac{\partial B}{\partial T_y^{lm}} &{} \frac{\partial B}{\partial S_x^{lm}} &{} \frac{\partial B}{\partial S_y^{lm}} &{} \frac{\partial B}{\partial \theta ^{lm}} \\ \frac{\partial C}{\partial T_x^{lm}} &{} \frac{\partial C}{\partial T_y^{lm}} &{} \frac{\partial C}{\partial S_x^{lm}} &{} \frac{\partial C}{\partial S_y^{lm}} &{} \frac{\partial C}{\partial \theta ^{lm}} \\ \frac{\partial D}{\partial T_x^{lm}} &{} \frac{\partial D}{\partial T_y^{lm}} &{} \frac{\partial D}{\partial S_x^{lm}} &{} \frac{\partial D}{\partial S_y^{lm}} &{} \frac{\partial D}{\partial \theta ^{lm}} \\ \frac{\partial E}{\partial T_x^{lm}} &{} \frac{\partial E}{\partial T_y^{lm}} &{} \frac{\partial E}{\partial S_x^{lm}} &{} \frac{\partial E}{\partial S_y^{lm}} &{} \frac{\partial E}{\partial \theta ^{lm}} \end{array} \right) \end{aligned}$$

15 $$\begin{aligned}&X = \left( \begin{array}{ccccc} \frac{\partial \varepsilon _i^2}{\partial T_{x}^{lm}} \\ \frac{\partial \varepsilon _i^2}{\partial T_{y}^{lm}} \\ \frac{\partial \varepsilon _i^2}{\partial S_{x}^{lm}} \\ \frac{\partial \varepsilon _i^2}{\partial S_{y}^{lm}} \\ \frac{\partial \varepsilon _i^2}{\partial \theta ^{lm}} \end{array} \right) =\left( \begin{array}{ccccc} A \\ B \\ C \\ D \\ E \end{array} \right) \end{aligned}$$

The iteration is terminated when all of the element of correction value $\Delta P$ becomes sufficiently small. Finally, the approximate solution thus obtained is substituted into Eq. (8) in order to be used for calibration of the coordinate system.

## Simulation

### Simulation setup

In this simulation, GMapping [22] for short-range Lidar SLAM and ORB-SLAM2 [23] for monocular SLAM was used. The PC used for the simulation had the following specifications. CPU: Intel Core i7-8700K @ 3.70GHz, memory: 64GB, OS: Ubuntu 18.04, and the system was built using a robot operating system (ROS) [24]. Gazebo [25] was used for evaluating the performance of the proposed method.

**Fig. 5:**
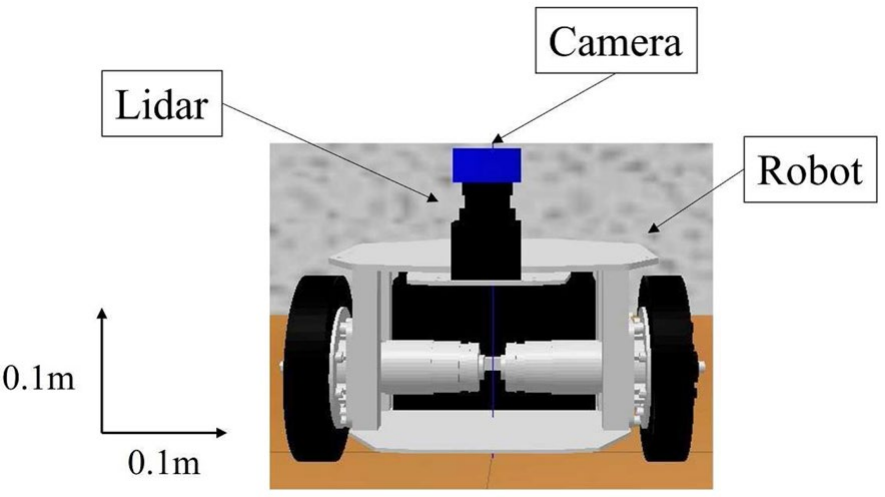
Robot and sensors

The robot and sensors used in Gazebo are shown in Table 1 and Fig. 5. The robot used is a two-wheel drive with rear casters. The measurement distance of the short-range Lidar was set to 4 m, measurement range to 270deg, resolution of the monocular camera to 640 480, and fps to 30.

**Table 1:** The specifications of sensors used for simulation

Sensor parameters	Value
(a) Specifications of Lidar	
Distance [m]	4
Angle [deg]	270
(b) Specifications of monocular camera	
Resolution	640 × 480
fps	30

**Fig. 6:**
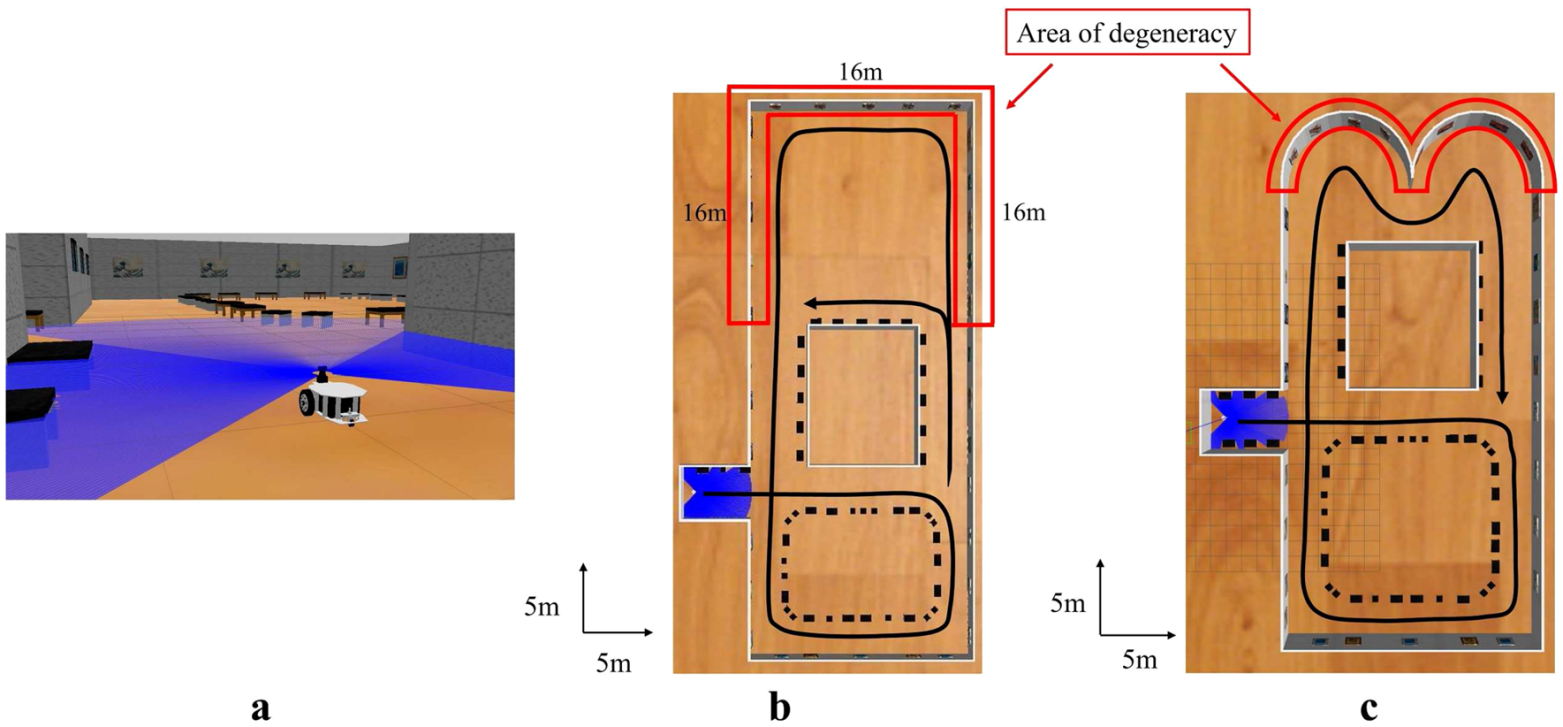
Simulation environment. **a** Robot view, **b** A top view of the environment for dealing with degeneracy in the direction of translation, **c** A top view of the environment for dealing with degeneracy in the direction of rotation

**Fig. 7:**
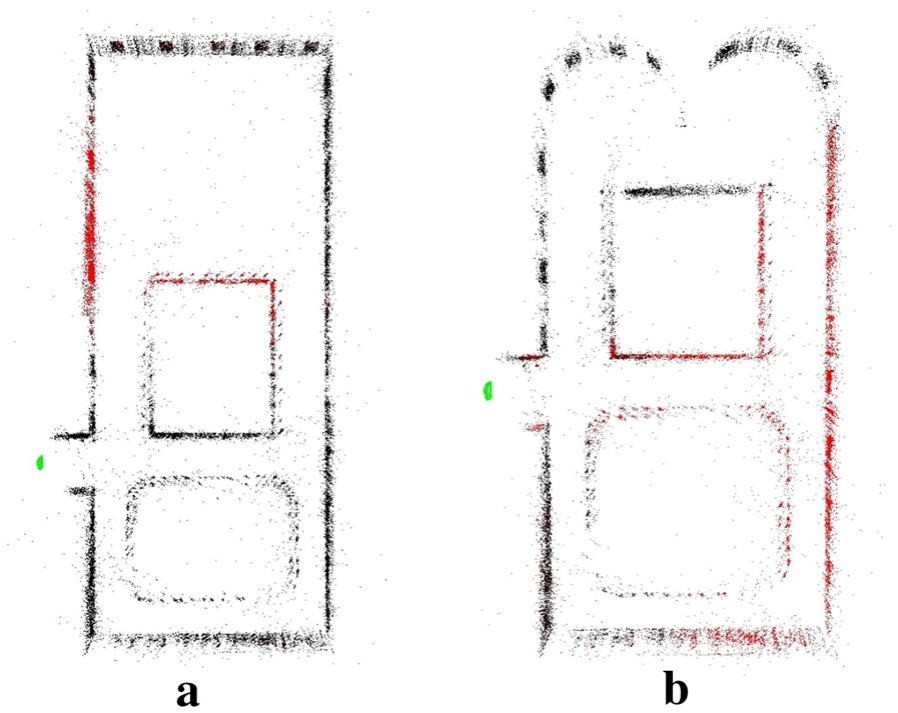
Feature-based map from monocular SLAM. **a** The environment for dealing with degeneracy in the direction of translation, **b** The environment for dealing with degeneracy in the direction of rotation

The simulated environment in Fig. 6 is assuming the museum and the mobile robot is expected to be used as guide robot. The blue line in Fig. 6a shows the visualization of the laser irradiation of Lidar. The environment in Fig. 6b represents the case the degeneracy occurs in the direction of translation, and that is Fig. 6c represents the case the degeneracy occurs in the direction of rotation. The robot was driven along the trajectory shown in black in Fig. 6b, c and the environmental map was built using the long-range Lidar, short-range Lidar, and proposed method, respectively. Here, the measurement distance of the long-range Lidar was set to 30 m. Moreover, the map in Fig. 7a, b was prepared in advance for the monocular localization.

In the environment shown in Fig. 6b, the robot first drives through the non-degenerate environment where the degeneracy does not occur, and then drives through the degenerate environment where degeneracy occurs in the short-range Lidar SLAM in the direction of translation (the area in the red box in Fig. 6b). Then it took the path of driving through the non-degenerate environment again. In the environment shown in Fig. 6c, the robot first drives through the non-degenerate environment as in Fig. 6b, and then performs rotational motion at a location where degeneracy occurs in the short-range Lidar SLAM in the direction of rotation (the area shown in the red frame in Fig. 6c). Then, it took the path of driving through the non-degenerate environment again.

In this simulation, *n* in Eq. (3) was set to 3, and degeneracy was considered to occur when $\Delta r_k$ is 0.1m or higher and $\Delta phi_k$ is 3deg or higher, based on a preliminary simulation results. The estimation of the transformation parameters begins when 30 or more corresponding poses are obtained, and the iterative calculation is terminated when all of the elements of the correction value $\Delta P=(\Delta T_x^{lm}$,$\Delta T_y^{lm}$,$\Delta S_x^{lm}$,$\Delta S_y^{lm}$,$\Delta \theta ^{lm})$ is less than 0.01.

### Simulation results and evaluation

**Fig. 8:**
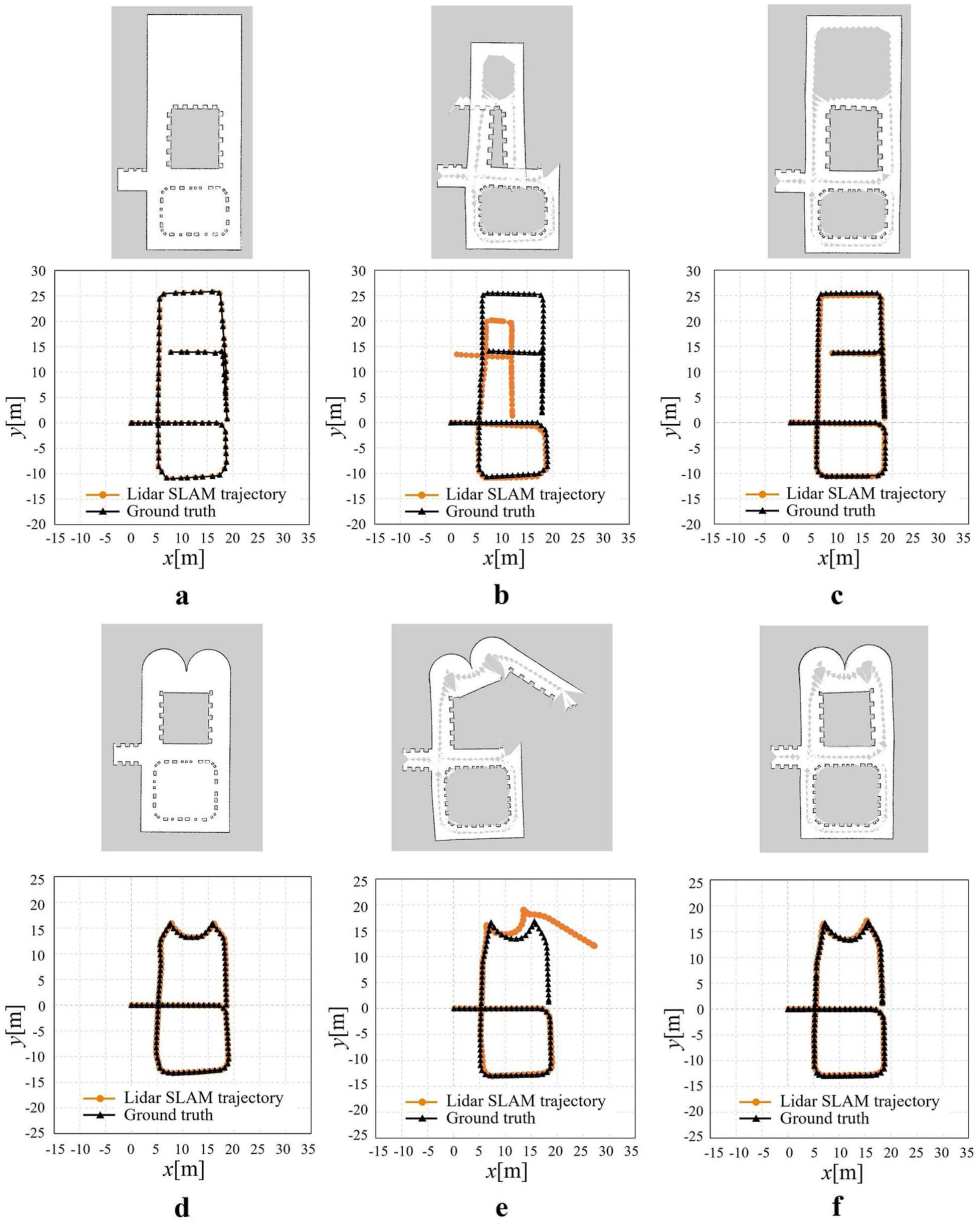
Environmental map and robot trajectory from Lidar SLAM. a, b, and c are the environment for dealing with degeneracy in the direction of translation; d, e, and f are the environment for dealing with degeneracy in the direction of rotation. **a** and **d** Measurement distance 30m, **b** and **e** Measurement distance 4m, **c** and **f** Measurement distance 4m (proposed method)

Figure 8 shows the created map and trajectory of the Lidar pose during the driving. Figure 8a, b, and c show the results obtained by the long range Lidar only, short range Lidar only, and the proposed method, respectively, when driving in the environment of Fig. 6b. Figure 8d, e, and f show those results when driving in the environment of Fig. 6c. In the built map, the white area indicates no obstacle, the black area indicates an obstacle, and the gray area indicates that the area has not been searched yet. The Lidar pose is plotted in orange, and the true value of the robot pose is plotted in black.

First, the results of simulations conducted in the Fig. 6b environment were as follows. The map built by the long-range Lidar SLAM, shown in Fig. 8a, was successful because the Lidar could measure the entire environment. However, the map built by the short-range Lidar SLAM did not match the wall on the right side, Fig. 8b. This is due to the degeneracy in the translation in the area in the red frame in Fig. 6b, resulting in a shrinking map. In contrast, Fig. 8c shows that the proposed method can maintain accuracy even in the degenerate environment. This is because degeneracy can be accurately predicted by the proposed system, and the pose supplemented by the calibrated pose prevents the map from shrinking.

The distance error between the Lidar pose and true value is shown in Table 2. Table 2 shows that the MAE (Mean Absolute Error) and RMSE (Root Mean Squared Error) values of the proposed method are smaller by 2.80 m and 3.86 m, respectively, compared to the short-range Lidar method, which confirms the improved localization accuracy of Lidar SLAM. Moreover, the MAE and RMSE of the proposed method were larger than those of the long-range Lidar by only 0.20 m and 0.25 m, respectively. Although the accuracy of the proposed method was slightly worse than that of long-range Lidar SLAM, it is expected that the proposed method can reach the accuracy of long-range Lidar with further improvements.

**Table 2:** Simulation results for dealing with degeneracy in the direction of translation

	Measurement distance 30m	Measurement distance 4m	Measurement distance 4m (proposed method)
MAE[m]	0.13	3.13	0.33
RMSE[m]	0.14	4.25	0.39
MAX[m]	0.25	8.18	1.29
MIN[m]	0.03	0.09	0.01

Next, the results of simulations conducted in Fig. 6c environment were as follows. The map built by the long-range Lidar SLAM, shown in Fig. 8d, was successful because the Lidar could measure the entire environment. However, the map built by the short-range Lidar SLAM is distorted as shown in Fig. 8e. This is due to the degeneracy in the direction of rotation in the area in the red frame in Fig. 6c, resulting in the reduced amount of rotation. In contrast, Fig. 8f shows that the proposed method can maintain accuracy even in the degenerate environment. This is because degeneracy can be accurately detected by the proposed system, and the pose supplement by the calibrated pose prevents the map from being distorted.

The distance error between the Lidar pose and true value is shown in Table 3. Table 3 shows that the MAE (Mean Absolute Error) and RMSE (Root Mean Squared Error) values of the proposed method are smaller by 1.50 m and 3.40 m, respectively, compared to the short-range Lidar method, which confirms the improved localization accuracy of Lidar SLAM. Moreover, compared with long-range Lidar, the MAE of the proposed method was smaller by 0.01 m, and the RMSE was equal to the proposed method, indicating that the accuracy of the proposed method is almost equivalent to that of long-range Lidar SLAM.

**Table 3:** Simulation results for dealing with degeneracy in the direction of rotation

	Measurement distance 30m	Measurement distance 4m	Measurement distance 4m (proposed method)
MAE[m]	0.20	1.69	0.19
RMSE[m]	0.21	3.61	0.21
MAX[m]	0.33	13.92	0.54
MIN[m]	0.05	0.02	0.02

Finally, further analysis of the internal behavior was conducted as follows.

**Fig. 9:**
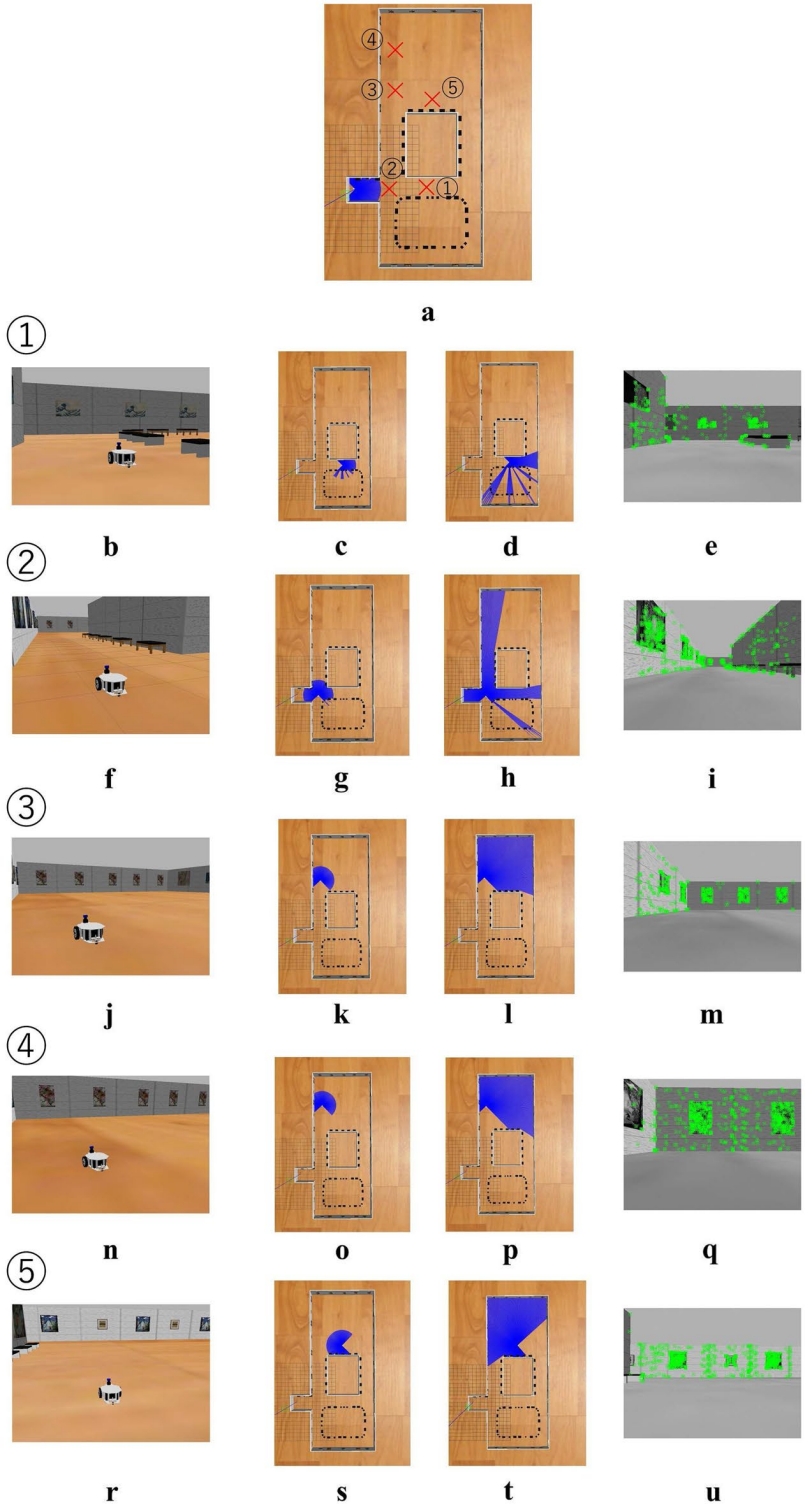
Overall landscape and sensor information in specific position along the robot trajectory in simulation environment dealing with degeneracy in the direction of translation. **a** Selected position, **b**, **f**, **j**, **n**, **r** Overall landscape of the robot and the environment, **c**, **g**, **k**, **o**, **s** Laser scans of 4m Lidar, **d**, **h**, **l**, **p**, **t** Laser scans of 30m Lidar, **e**, **i**, **m**, **q**, **u** Images from monocular camera

First, we start from the case of simulation in Fig. 6b environment. Figure 9 shows the overall landscape of the robot and the environment, sensor information (laser scans) from the short-range Lidar SLAM and the long-range Lidar SLAM, and sensor information (images from the monocular camera) from the monocular localization at a few specific locations along the movement trajectory (indicated by in Fig. 9a). In particular, two locations are selected from the area where degeneracy occurs in order to focus on the situation where degeneracy occurs.

**Fig. 10:**
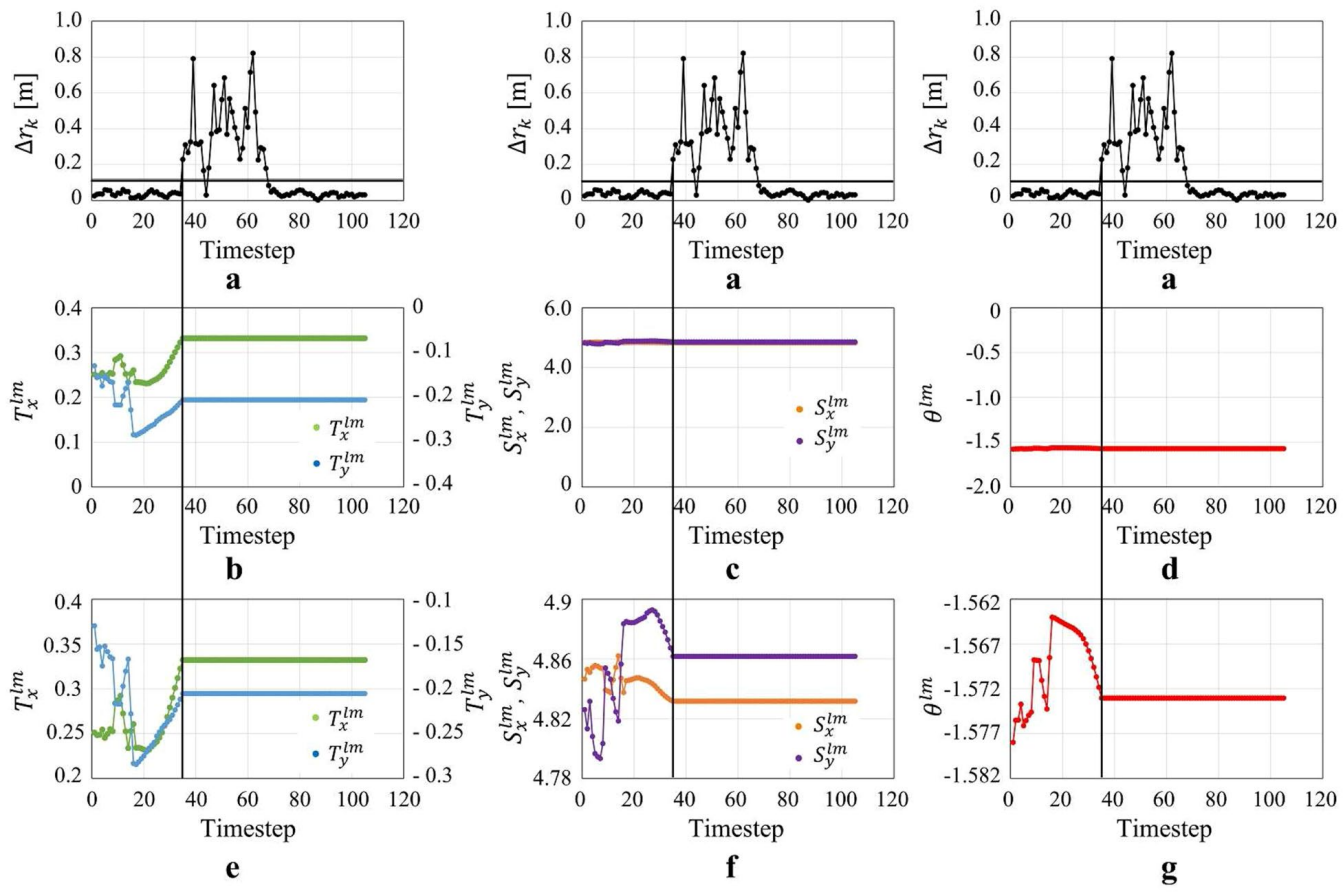
Transition of the degeneracy detection index and transformation parameters in case of simulation dealing with degeneracy in the direction of translation. **a** degeneracy detection index, **b**, **e** Parameter transition of $T_x^{lm}$ and $T_y^{lm}$, **c**, **f** Parameter transition of $S_x^{lm}$ and $S_y^{lm}$, **d**, **g** Parameter transition of $\theta ^{lm}$

Figure 10 shows the transition of the degeneracy detection index $\Delta r_k$ and the transformation parameters. Figure 12a shows the results of degeneracy detection at each location along the trajectory. The location inside the red frame in Fig. 6b, which is considered as the environment where degeneracy occurs, correponds to the area in the red frame in Fig. 12a, indicating that degeneracy occurs over a long period of time. The detail situation where the degeneracy occurs could be seen in Fig. 9. From Fig. 9k, 9o, it can be seen that the laser scan of the short-range Lidar has not changed in this area in terms of movement. This is a situation where pose translation cannot be estimated from the laser scan, which is nothing but degeneracy. On the other hand, from Fig. 9l, 9p and Fig. 9m, 9q, the laser scan of the long-range Lidar and the image of the monocular camera change with movement indicates that no degeneracy is occuring even in this area.

**Fig. 11:**
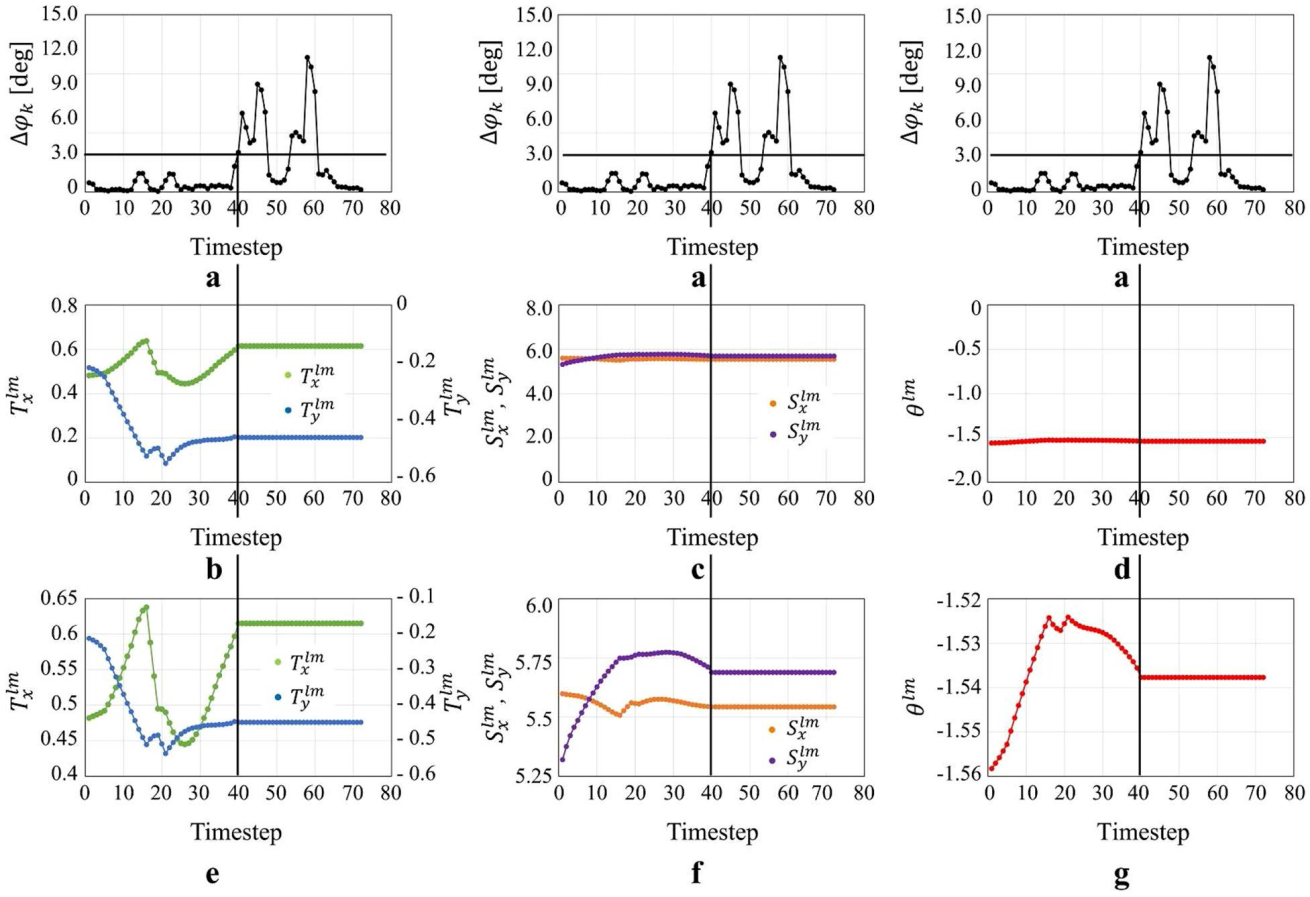
Transition of the degeneracy detection index and transformation parameters in case of simulation dealing with degeneracy in the direction of rotation. **a** Degeneracy detection index, **b**, **e** Parameter transition of $T_x^{lm}$ and $T_y^{lm}$, **c**, **f** Parameter transition of $S_x^{lm}$ and $S_y^{lm}$, **d**, **g** Parameter transition of $\theta ^{lm}$

**Fig. 12:**
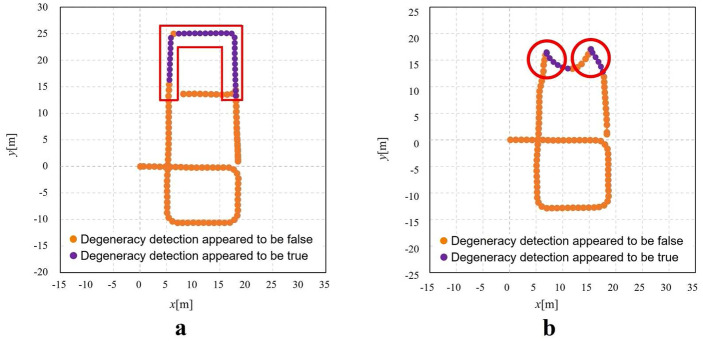
Result of degeneracy detection at each location **a** Result in the environment for dealing with degeneracy in the direction of translation, **b** Result in the environment for dealing with degeneracy in the direction of rotation

Next is the case of simulation conducted in Fig. 6c environment. Figure 11 shows the transition of the degeneracy detection index $\Delta \phi _k$ and the transformation parameters. Figure 12b shows the results of degeneracy detection at each location along the trajectory. The location inside the red frame in Fig. 6c, which is considered as the degenerate environment, corresponds to the area in the red frame in Fig. 12b, indicating that degeneracy occurs at those points.

Therefore, the proposed method, which automatically detects degeneracy immediately and switches to the calibrated pose based on the pose information from the monocular localization with higher accuracy, is expected to work in these situations.

## Conclusion

In this study, we proposed a short-range Lidar SLAM that utilizes localization data from the monocular localization as a supplement, with the goal of realizing a high-accuracy SLAM with low-cost sensors in environments where degeneracy occurs. Then, the accuracy of the proposed short-range Lidar SLAM was evaluated via a simulation in an environment where degeneracy occurs. The MAE and RSME of the distance error between Lidar position and true value was compared in three cases, that is, long-range Lidar (ideal case), short-range Lidar, and proposed method. The results showed that the MAE and RSME significantly decreased from that of normal short-range Lidar SLAM and was close to the level of long-range Lidar SLAM. Therefore, the effectiveness of the method was verified to certain extent.

As a future work, we would like to conduct experiments in an actual environments using an actual robot.

## Data Availability

Not applicable.

## Abbreviations

SLAM: Simultaneous localization and mapping
ROS: Robot operating system

## References

[1] Dissanayake G, Newman P, Clark S, Durrant-Whyte H, Csorba M (2001). A solution to the simultaneous localisation and map building (slam) problem. IEEE Trans Robot Autom.

[2] Zhang Y, Zhang H, Xiong Z, Sheng X (2019) A visual slam system with laser assisted optimization. In: Proceedings of the 2019 IEEE/ASME International Conference on Advanced Intelligent Mechatronics, pp. 187–192

[3] Shin Y, Park Y, Kim A (2018) Direct visual slam using sparse depth for camera-lidar system. In: Proceedings of the 2018 IEEE International Conference on Robotics and Automation, pp. 5144–5151

[4] Park K, Kim S, Sohn K (2020). High-precision depth estimation using uncalibrated lidar and stereo fusion. IEEE Trans Intell Transport Syst.

[5] Pandey G, Savarese S, McBride J, Eustice R (2011) Visually bootstrapped generalized icp. In: Proceedings of the 2011 IEEE International Conference on Robotics and Automation, pp. 2660–2667

[6] Jiang G, Yin L, Jin S, Tian C, Ma X, Ou Y (2019). A simultaneous localization and mapping (slam) framework for 2.5d map building based on low-cost lidar and vision fusion. Appl Sci.

[7] Zhang Z, Zhao R, Liu E, Yan K, Ma Y (2018). Scale estimation and correction of the monocular simultaneous localization and mapping (slam) based on fusion of 1d laser range finder and vision data. Sensors.

[8] Sun F, Zhou Y, Li C, Huang Y (2010) Research on active slam with fusion of monocular vision and laser range data. In: Proceedings of the 2010 8th World Congress on Intelligent Control and Automation, pp. 6550–6554

[9] Graeter J, Wilczynski A, Lauer, M.: Limo, (2018) Limo: Lidar-monocular visual odometry. In: Proceedings of the 2018 IEEE/RSJ International Conference on Intelligent Robots and Systems, pp. 7872–7879

[10] Ramos F, Fox D, Durrant-Whyte H (2007) Crf-matching: Conditional random fields for feature-based scan matching. In: Proceedings of the Robotics: Science and Systems, pp. 27–30

[11] Oh T, Lee D, Kim H, Myung H (2015). Graph structure-based simultaneous localization and mapping using a hybrid method of 2d laser scan and monocular camera image in environments with laser scan ambiguity. Sensors.

[12] Zhu Z, Yang S, Dai H, Li F (2018) Loop detection and correction of 3d laser-based slam with visual information. In: Proceedings of the 31st International Conference on Computer Animation and Social Agents, pp. 53–58

[13] Liang X, Chen H, Li Y, Liu Y (2016) Visual laser-slam in large-scale indoor environments. In: Proceedings of the 2016 IEEE International Conference on Robotics and Biomimetics, pp. 19–24

[14] Chan S, Wu P, Fu L (2018). Robust 2d indoor localization through laser slam and visual slam fusion. In: Proceedings of the 2018 IEEE International Conference on Systems, Man, and Cybernetics, pp. 1263–1268

[15] Debeunne C, Vivet D (2020) A review of visual-lidar fusion based simultaneous localization and mapping. Sensors 4, 2068–2087 10.3390/s20072068 PMC7181037 32272649

[16] Biber P, Strasser W (2003) The normal distributions transform: A new approach to laser scan matching. In: Proceedings of the 2003 IEEE/RSJ nU. Conference on Intelligent Robots and Systems, pp. 2743–2748

[17] Zhang J, Kaess M, Singh S (2016) On degeneracy of optimization-based state estimation problems. In: IEEE International Conference on Robotics and Automation, pp. 809–816

[18] Ebadi K, Palieri M, Wood S, Padgett C, Agha-mohammadi A (2021) Dare-slam: Degeneracy-aware and resilient loop closing in perceptually-degraded environments. In: Intelligent and Robotic Systems, p. 2

[19] Umeyama S (1991) Least-squares estimation of transformation parameters between two point patterns. In: IEEE Transactions on Pattern Analysis and Machine Intelligence, pp. 376–380

[20] Zhang, Z., Scaramuzza, D.: A tutorial on quantitative trajectory evaluation for visual(-inertial) odometry. In: IEEE International Workshop on Intelligent Robots and Systems, pp. 7244–7251 (2018)

[21] Sasaki T, Hashimoto H (2008) Automated calibration of distributed laser range finders based on object tracking in overlapping sensing regions. In: Proceedings of the 17th World Congress the International Federation of Automatic Control, pp. 8203–8208

[22] Grisetti G, Stachniss C (2007). Improved techniques for grid mapping with rao-blackwellized particle filters. IEEE Trans. on Robotics.

[23] Mur-Artal R, Tardos J (2017). Orb-slam2: an open-source slam system for monocular, stereo and rgb-d cameras. IEEE Trans Robot.

[24] Quigley M, Gerkey B, Ken C, Faust J, Foote T, Leibs J, Berger E, Wheeler R, Ng, A.: Ros, (2009) Ros: An open-source robot operating system. In: Proceedings of the 2009 IEEE International Conference on Robotics and Automation, Workshop on Open Source Software, pp. 1–6

[25] Koenig N, Howard A (2004) Design and use paradigms for gazebo, an open-source multi-robot simulator. In: Proceedings of the 2004 IEEE/RSJ International Conference on Intelligent Robots and Systems, pp. 2149–2154

